# Luminescence of Agrotextiles Based on Red-Light-Emitting Organic Luminophore and Polypropylene Spunbond Enhances the Growth and Photosynthesis of Vegetable Plants

**DOI:** 10.3389/fpls.2022.827679

**Published:** 2022-04-21

**Authors:** Robert Khramov, Anatoly Kosobryukhov, Vladimir Kreslavski, Dmitry Balakirev, Alexandra Khudyakova, Evgeniya Svidchenko, Nikolay Surin, Sergey Ponomarenko, Yuriy Luponosov

**Affiliations:** ^1^Institute of Theoretical and Experimental Biophysics, Russian Academy of Sciences, Pushchino, Russia; ^2^Institute of Basic Biological Problems, Russian Academy of Sciences, Pushchino, Russia; ^3^Enikolopov Institute of Synthetic Polymeric Materials of the Russian Academy of Sciences, Moscow, Russia; ^4^Chemistry Department, Moscow State University, Moscow, Russia

**Keywords:** agrotextile spunbond, luminescent polylactide, plants photobiomodulation, red organic photoluminophore, photosynthesis, polymer non-woven agrotextile, plant growth

## Abstract

The impact of a light-transforming covering on photosynthetic activity and growth processes in lettuce and white cabbage plants grown in a glass greenhouse was studied. Plants were covered with agrotextile, a polypropylene (PP) nonwoven spunbond coated with polylactide varnish containing a new organic luminophore (LUM), which absorbs sunlight mainly in the 460–560 nm region and efficiently reradiates it in the red spectral region with a maximum at 660 nm. For comparison, simultaneously two references agrotextiles without LUM or containing a non-luminescent chromophore (ABS) with an absorption spectrum close to that of LUM were as well investigated. The use of the agrotextile with LUM resulted in a significant increase in total crude aboveground biomass for 32-, 33-, and 43-day-old plants on the average by 20–40%, and the photosynthesis rate increased on the average by 30–40% compared to the agrotextile without LUM. The use of the agrotextile with ABS mimicking the absorption of LUM also did not reveal a significant impact on photosynthesis and biomass accumulation in the plants as compared to the reference agrotextile coated only with the polylactide varnish. At the same time, the photosystem II activity (*F*_v_/*F*_m_ and *F*′_v_/*F*′_m_ quantum yields) was nearly the same in all experiments. When plants were grown under the light-converting agrotextile, the luminescent component of the converted light in the red spectrum region led to an increase in plant growth and photosynthesis rate, which is a fundamentally new result. Possible reasons for the stimulation of growth and photosynthesis due to the redistribution of the light spectral composition were analyzed. The use of covering materials containing luminophores similar to LUM can be promising in agrobiotechnology not only for green and vegetable crops but also for other field and greenhouse crops and various fruit bushes and trees.

## Introduction

Polymeric materials of various types are widely used in agriculture as a covering for the cultivation of plants in various facilities. The durability, the maintenance of higher temperatures under coverings in cold weather, and the possibility to optimize the parameters of irradiation of plants in the UV, visible and near infrared spectral regions are important characteristics of these materials (Kusnetsov et al., 1989; Edser, 2002; Espi et al., 2006; Lamnatou and Chemisana, 2013). At the same integral intensity of solar radiation, an increase in the light fraction in the red spectrum region may lead to an increase in the rate of photosynthesis (Ohasi-Kaneko et al., 2007), whereas green light is less efficient for photosynthesis than red and blue light. Therefore, it is important to redistribute ineffective or excess components of solar radiation in favor of more efficient components (usually red and near-IR light) to maintain the function of living organisms (Kosobryukhov et al., 2000; Khramov et al., 2010, 2015, 2019). Excessive solar UV radiation can damage various cellular systems, leading to additional energy costs for their restoration and inhibit plant growth and photosynthesis (Tsormpatsidis et al., 2010; Kosobryukhov et al., 2020).

The active development and research of new covering films with photoluminophores (PLs) to optimize light conditions for plant growth began at the end of the last century (Kusnetsov et al., 1989). Initially, UV-absorbing organic and later inorganic fluorescent compounds, including complexes of rare-earth metals, were used for this purpose (Bratkova et al., 1998; Bratkova and Schelokov, 1999). The introduction of these compounds into a covering polyethylene film for greenhouses resulted in an increase in the photosynthesis rate and plant growth, and ultimately in the plant productivity. Interestingly, additional PL emission little affects the intensity of solar radiation incident on plants in the region of orange–red light. The most frequently used PLs, upon excitation in the UV region of solar radiation, show fluorescence with a maximum at 611–625 nm both in traditional polymer films (Raida et al., 2004; Vorobiev et al., 2010; Yuan et al., 2014; Yang et al., 2018) and in modified covering nonwoven materials (Khramov et al., 2019). It should be noted that the positive effect of coverings with introduced PLs depends on the intensity of the UV component in solar radiation. This may be one of the reasons for the dependence of the effects of enhancing the plant growth on weather conditions characterized by different levels of UV radiation. Since the fraction of UV radiation being transformed is small, attempts are made to obtain PLs based on rare-earth metals with an excitation spectrum in a more intense blue–green spectral region (Lian et al., 2002) or to use efficient organic PLs (Loik et al., 2017; Sánchez-Lanuza et al., 2021) and colloidal nanocrystals (Simakin et al., 2020). Nevertheless, an analysis of the current state of art in this field (Minich et al., 2006, 2011; Zhao et al., 2014; Khramov et al., 2019; Simakin et al., 2020; Wang et al., 2020) showed few examples of covering materials with PLs that effectively emit light in the red spectral region. As a rule, their luminescence maximum is usually in the region of 611–625 nm, which is far from the region of 650–680 nm, the maximum in the absorption spectrum of chlorophylls, and 660–670 nm, the maximum in the absorption spectrum of phytochrome.

In this regard, significant advances may be achieved by the further development of PLs with emission in the red region and excitation in the yellow–green spectral region, which is characterized by a lower photosynthesis intensity compared with the region of red light in the action spectrum of photosynthesis (McCree, 1972). PLs based on organic conjugated donor–acceptor molecules with triphenylamine fragments are one of the most promising classes of luminophores for this purpose (Balakirev et al., 2020; Luponosov et al., 2020). In this case, it is important to estimate the influence of changes in the light spectral composition on photosynthesis at both the primary light and secondary dark stages.

Therefore, in this work, special attention was paid to elucidating the influence of various spectral components of converted solar radiation on the constituents of the production process in plants, namely, photosynthesis, respiration, and growth. At the same time, by using the organic red-light-emitting PL, we transformed the solar radiation of the blue–green region into the radiation of the red region to increase the fraction of red light in photosynthetically active radiation.

The aim of the present work was to study the influence of solar radiation passed through a common white polypropylene (PP) spunbond modified by an organic red-light-emitting PL on the growth, the rate of photosynthesis and transpiration, stomatal conductance, and water use efficiency (WUE) in white cabbage and lettuce plants at early developmental stages and to analyze the advantages of these technologies.

## Materials and Methods

### Experimental Scheme and Plant Cultivation

**Figure 1:**
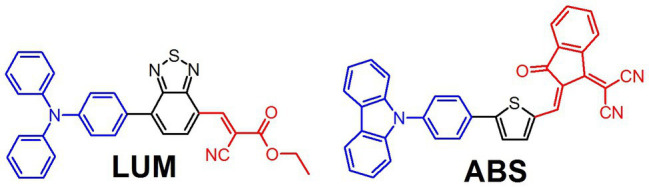
Chemical structures of the organic luminophore used in this study (LUM) and the nonluminescent chromophore absorbent (ABS).

**Figure 2:**
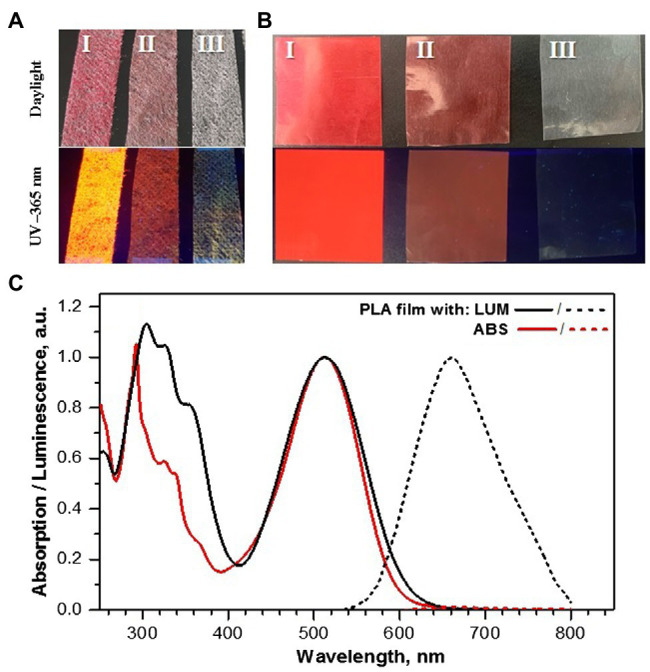
**(A–C)** Photographs of **(A)** agrotextiles (pieces 2 × 5 cm in size) and **(B)** pressed PLA films (3 × 3 cm) under daylight and irradiation with UV = 365 nm: I—with LUM (w/w 0.25%); II—with ABS (w/w 0.25%); III—reference sample, PP + PLA textile, and PLA film, respectively. **(C)** Normalized absorption (solid) and luminescence (dashed) spectra of PLA films (thickness is *ca*. 40 μm) containing (w/w 0.25%) luminophore LUM or absorbent ABS.

In the experiments, we used the organic photoluminophore (PL) LUM (Figure 1). LUM shows a bright red emission with a luminescence maximum at 660 nm and a high photoluminescence quantum yield (PLQY) in the polymer matrix. To estimate the influence of the PL absorption on the growth of plants under light-converting agrotextiles, we synthesized a model non-luminescent chromophore ABS (Figure 1) with an absorption spectrum similar to that of LUM (Figure 2). The factors influencing the growth when using coatings were also evaluated and analyzed: photosynthesis, respiration, and the balance of energy and organic matter were calculated, which were determined from the difference between the rates of photosynthesis and respiration of plant leaves.

Plants were grown in Pushchino (Moscow Region, Russia). The Geocoordinates of the greenhouse are: latitude: 54°50′01″ N and longitude: 37°36′40″ E. The light-converting material can exert different effects on plants, depending on the ambient temperature and the level of solar radiation, so it was important to test the coatings under various environmental conditions during the spring–summer period.

Testing of the light-converting agrotextiles was carried out during May–August in a glass greenhouse to create stable environmental conditions and splited into two series of subsequent experiments with the same agrotextiles. In the first series of experiments carried out in May–June, the following coverings were used: (1) commercial non-modified polypropylene spunbond (PP); (2) PP spunbond coated with polylactide (PLA) varnish (PP + PLA); (3) PP spunbond coated with PLA varnish containing photoluminophore LUM (PP + PLA + LUM); and (4) PP spunbond coated with PLA varnish with the addition of chromophore ABS (PP + PLA + ABS). It should be noted that we did not find reliable differences in any parameters for plants between PP and PP + PLA coverings. Therefore, we further focused on comparison parameters for three coverings: PP + PLA + LUM, PP + PLA + ABS, and PP + PLA. The average light intensity in the greenhouse measured at noon was 950 ± 90 μmol quanta m^−2^s^−1^. The light intensity under coverings with PL was lower than under coverings without PL by an average by 15–20%. The temperature in the greenhouse was in the range of 24–30/18–23°C for day/night.

In the second series of experiments carried out in July–August, the same agrotextiles were used as in the first series. At the same time, the average light intensity in the greenhouse due to better weather conditions was slightly higher than that in the first series of experiments: 1100 ± 100 μmol quanta m^−2^s^−1^. The day/night temperature regime in the greenhouse was (26–32)/(20–26)°С.

White cabbage plants of the early variety, cultivar “Parel F1,” and lettuce plants, cultivar “Kucheryavets Odesskiy,” were grown in plastic vessels with a height and a diameter of 10 cm filled with nutrient soil for seedlings. The soil contained 250 mg/kg of nitrogen, 400 mg/kg of phosphorus, and 500 mg/kg of potassium, and the pH of the soil was 6.5. The normal water regime was maintained by daily watering of plants. The coverings were fixed horizontally at a level of 30–35 cm above the plants placed on laboratory tables. The side spaces between the surface of the tables and the covers were free for air convection, which ensured almost the same temperature regime for all coverings.

Initially, each series contained 32–36 seedlings of cabbage and lettuce. Then, for each species, approximately 20–24 plants of the same size were selected and grown for 32, 33, or 43 days. The accumulation of plant biomass was measured at Days 33 and 43 from the beginning of the first experiment and at Days 16 and 32 in the second experiment.

### Synthesis of LUM and ABS

The synthesis of LUM and ABS and their detailed characterization are described in the Electronic Supplementary Information (ESI). The chemical structure and high purity of LUM and ABS were confirmed by the complex of methods (Supplementary Figures S1–S5).

### Preparation of Light-Converting Films and Agrotextiles

PLA films with side lengths 5 × 5 cm and 40 μm thickness containing 0.25% (*w/w*) LUM or ABS were obtained by a solution method from chloroform followed by a hot matrix pressing (5 min, 240°C, 100 kg/cm^2^). The standard procedure of the polymer varnish preparation includes dissolution of PLA 4032D (8.00 g) and LUM or ABS (120.0 mg; *w/w* 0.25%) in 830 ml of chloroform at 60°C. Afterwards, the resulting homogeneous varnish solution was deposited onto the commercial nonwoven PP spunbond [averaged density is 20 g/m^2^; piece size 120 × 150 cm (1.8 m^2^)] using a polyamide fiber roller. The target PP agrotextiles (PP + PLA + LUM and PP + PLA + ABS) contain *ca*. 17.5 ± 0.2% (*w/w*) of PLA with 0.25 ± 0.02% (*w/w*) LUM or ABS. The control blank Spunbond (PP + PLA) contains *ca*. 17.5 ± 0.2% (*w/w*) of PLA.

### Methods for the Study of Plants

The Photosystem II (PS2) maximum and (*F*_v_/*F*_m_) and effective (*F*′_v_/*F*′_m_) quantum yields were determined by modulated pulse methods using a JUNIOR-PAM fluorometer (Walz, Germany). Photoinduced changes in fluorescence were calculated according to the equation: (*F*_v_) = *F*_m_ – *F*_0_, where *F*_0_ is the initial level of fluorescence, and *F*_m_ is the maximum level of fluorescence; these values were determined after 15 min dark adaptation of the leaves. Photoinduced fluorescence changes under light saturation conditions were calculated according to the equation: (*F′*_v_) = *F*′_m_ – *F′*_0_, where *F′*_m_ and *F′*_0_ are the maximum and initial fluorescence levels, respectively.

The photosynthesis and respiration rates, stomatal conductance, transpiration rate, and WUE were determined under daylight conditions using an LCPro+ portable infrared gas analyzer (ADC BioScientific Ltd., United Kingdom) connected to a leaf chamber with an area of 6.25 cm^2^. The measurements were carried out in the morning, usually between 9 and 11 am, with an average light intensity of 450 ± 45 μmol photons m^−2^s^−1^. The upper fully developed leaves were used for the analysis. The content of Chl *a* and Chl *b* and carotenoids was measured using the method as described previously (Lichtenthaler and Wellburn, 1987). Sample absorption was measured in ethanol extracts using a spectrophotometer (Genesis 10UV, Thermo Spectronic, United States) at wavelengths of 470, 649, and 665 nm.

#### Absorption and Luminescence Spectroscopy

The absorption spectra were recorded on a Shimadzu UV-2501PC spectrophotometer (Japan). Photoluminescence spectra were recorded in 200-700 nm region using a scanning spectrofluorimeter ALS01 M (Russia) with registration in the single photon counting mode at successive time intervals and automatic adjustment of emission intensity. Photoluminescence spectra in the PLA matrix and non-woven coverings were measured in the integrating sphere. The photoluminescence quantum yield of the materials was measured relative to the 2,1,3-benzothiadiazole-based luminophores in the polystyrene matrix (Skorotetcky et al., 2018).

#### Solar Irradiance

The solar irradiance spectra before and after passing through different types of the agrotextile were measured in the wavelength range of 380–780 nm at a clear sky using a “MK350N Premium” spectroradiometer (Taiwan). The measurements were carried out with the direction of the sensitive (measuring) window of the radiometer toward the zenith.

**Figure 3:**
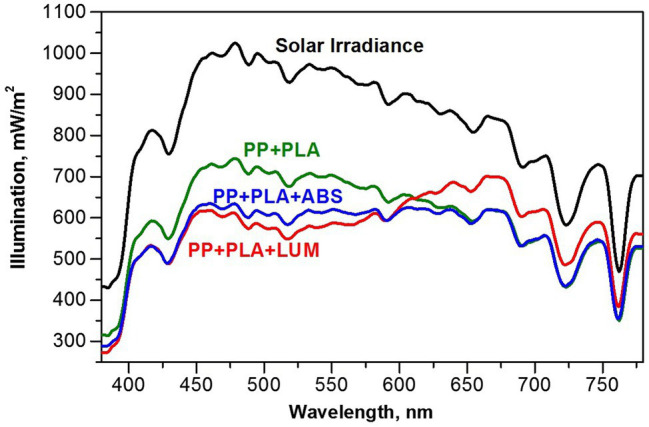
Changes in the absorption spectrum of solar light (Solar Irradiance) after passing through the reference PP + PLA textile and agrotextiles with addition (w/w 0.25%) of the LUM (PP + PLA + LUM) or ABS (PP + PLA + LUM).

Changes in the spectral distribution of the solar radiation under different agrotextiles are presented in Figure 3 and summarized in Table 1.

**Table 1:** Percent contribution of light of different spectral regions: blue (BL), orange–red (ORL), and yellow–green (YGL) inside (above and below the coverings) and outside of the greenhouse during the cultivation period.

Series/spectral region	Outside the greenhouse	Above covers	PP + PLA	PP + PLA+ LUM	(Exp-Cont)/Cont,%
BL (400–500 nm)	26.6 ± 0.5	27.1 ± 1.0	26.4 ± 0.8	22.0 ± 0.5	−16
YGL (500–600 nm)	35.9 ± 0.2	36.4 ± 0.1	36.4 ± 0.2	31.2 ± 0.6	−14
ORL (600–700 nm)	37.5 ± 0.4	36.5 ± 0.9	37.2 ± 0.7	46.8 ± 0.2	+26

Ratios for PP + PLA (Cont) and PP + PLA + LUM (Exp) below coverings are given. The results are given as % of the total photosynthetically active radiation (PAR) intensity. The averaged data of measurements (*n* = 20) performed at noon on cloudless days at an average solar radiation intensity in a glass greenhouse of 1,420 ± 157 μmol quanta m^−2^s^−1^, as well as on cloudy days at an average light intensity of approximately 294 ± 35 μmol quanta m^−2^s^−1^ are shown. The relative changes in a sample compared to the control are presented as (Samp-Cont)/Cont, %.

### Statistics

At least 10 cabbage plants and 12 lettuce plants were used for each series of measurements of growth parameters. The rates of photosynthesis and respiration as well as the leaf area were measured using 5–10 plants for each series. Fluorescence parameters were measured using 8–10 leaf disks with a diameter of 1 cm.

The arithmetic means and SDs were determined. The significance of differences between two series was described by the *t*-test at the 5% significance level. A comparison of the data for three groups of plants was performed by one-factor ANOVA and the Tukey’s multiple comparison test.

## Results

### Optical Properties of PP Spunbonds Modified With LUM and ABS

Due to the poor compatibility of polar organic donor-acceptor PLs with the non-polar polypropylene matrix, it was proposed to modify the commercial non-woven PP spunbond with a PLA coating containing photoluminophore LUM to obtain light-converting agrotextiles based on PP. The resulted agrotextiles PP + PLA + LUM and PP + PLA + ABS with an area of 1.8 m^2^ and a total mass concentration of LUM or ABS of *ca*. 0.25%, were compared to a model PP agrotextile coated with PLA only (Figure 2A). Since optical characteristics of the LUM and ABS in the agrotextiles obtained should be close to those in the PLA matrix, their optical properties were studied in detail in PLA-based films with a similar concentration of LUM or ABS (*w/w* 0.25%; Figure 2B). The UV–VIS absorption and luminescence spectra of PLA-based films with LUM and ABS are shown in Figure 2C.

As seen from the UV–VIS absorption and luminescence spectra, the shape of the absorption spectra for LUM and ABS is very close to each other. The both compounds have several bands of different intensity in UV spectral region, which can be ascribed to π-π* transitions and the intensive band in blue-green spectral region usually ascribed in such donor-acceptor molecules to the intermolecular charge transfer (Balakirev et al., 2020; Luponosov et al., 2020). In PLA matrix, the LUM shows intensive luminescence in the 600–720 nm range with the maximum at 660 nm and a high PLQY (40 ± 5%). In contrast, the ABS practically does not have any luminescence. The absorption spectrum of the PP + PLA + ABS and PP + PLA + LUM agrotextiles is not discussed due to strong light scattering processes. The recorded luminescence spectrum of PP + PLA + LUM (not shown) was in the same region as for the PLA-based film of the LUM.

Solar radiation spectra recorded outside and inside the greenhouse (above and below coverings), as well as under the model PP + PLA, showed no significant difference in energy redistribution in the blue, yellow–green, and orange–red regions of the sun spectrum (Table 1). However, the light intensity in case of PP + PLA + LUM was found to be increased in the orange–red region by 26%, while light intensity in the yellow–green and blue regions decreased by 14 and 16%, respectively, as compared to the model PP + PLA textile. The increase in light intensity in the region of 600–780 nm is due to the efficient luminescence contribution of the LUM, whereas the emission of PP + PLA + ABS covering in this spectral region is absent. Thus, PP + PLA + LUM is an example of light-converting PP agrotextile with a noticeable redistribution of sunlight in the visible region.

### Plant Growth and Photosynthesis

It should be noted that, in all cases, all physiological parameters of the plants under PP and PP-PLA coverings did not significantly differ from each other. Therefore, we further focused on comparison parameters for other three coverings: PP + PLA + LUM, PP + PLA + ABS, and PP + PLA.

**Figure 4:**
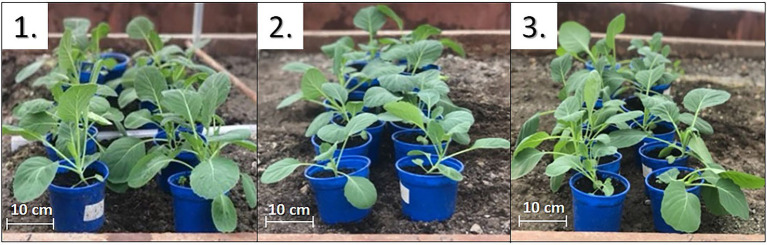
Cabbage plants grown for 33 days under different agrotextiles, photos 1–3 correspond to plants grown under agrotextiles: 1—PP + PLA + LUM; 2—PP + PLA; and 3—PP + PLA + ABS.

**Figure 5:**
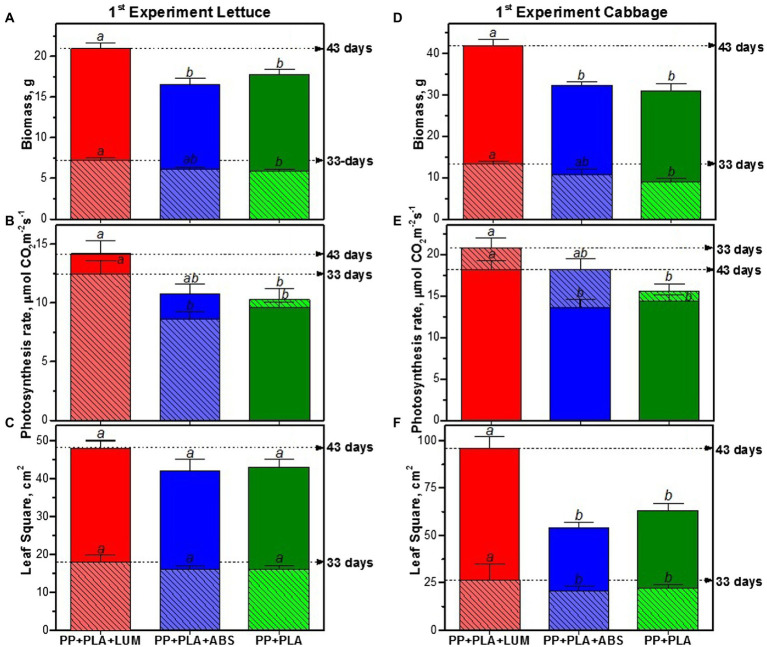
Experiment 1. Biomass accumulation **(A,D)**, photosynthesis rate **(B,E)**, and average leaf area **(C,F)** of lettuce and cabbage, respectively, grown for 33 days (columns marked with light tone and shading) and 43 days (standard columns) under agro textiles: PP + PLA + LUM, PP + PLA + ABS, and PP + PLA. Mean values ± SE are given. The values indicated in a row by different letters (a or b) significantly differ from each other (*p* < 0.05).

The usage of LUM as a red-light-emitting photoluminophore in a light-converting agrotextile covering material based on polypropylene (PP + PLA + LUM) leads to an increase in the rate of leaf photosynthesis and the accumulation of plant biomass (Figures 4, 5).

In the first series of experiments, the biomass of the lettuce increased by 33rd and 43rd days by 20 ± 3%, the photosynthesis rate increased by 27 ± 6%, and the increase in the leaf surface area did not exceed 12% (Figures 5A–C). The biomass accumulation by cabbage plants increased by 42 ± 7% compared to the series without the photoluminophore (Figure 5D). In the plants, under the light-transforming material, the photosynthesis rate per unit leaf area was 27 ± 6% higher (Figure 5E), and the change in the area was 48 ± 4% (Figure 5F).

**Figure 6:**
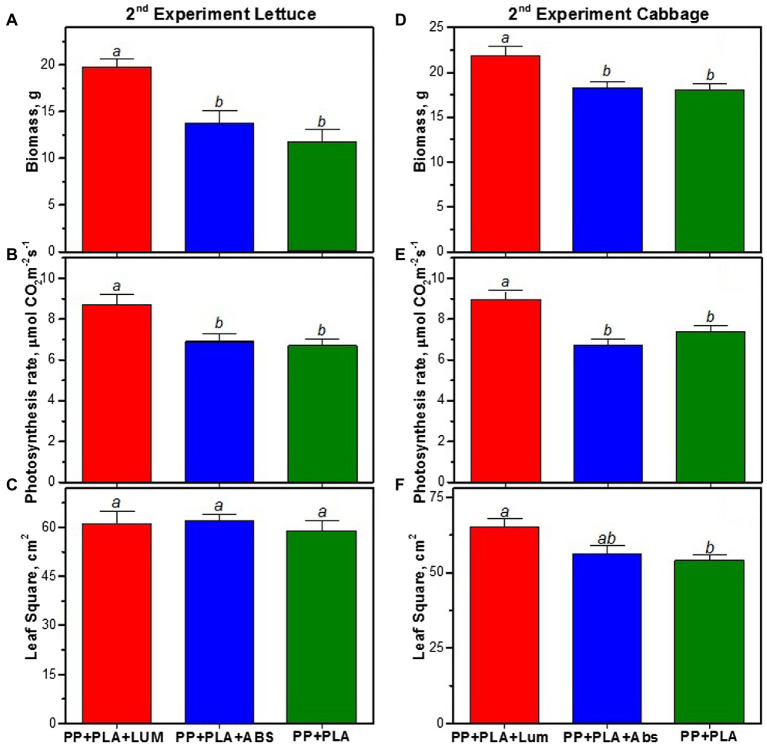
Experiment 2. Biomass accumulation **(A,D)**, photosynthesis rate **(B,E)**, and average leaf area **(C,F)** of lettuce and cabbage, respectively, grown for 32 days under agrotextiles: PP + PLA + LUM, PP + PLA + ABS, and PP + PLA. Mean values ± SE are given. The values indicated in a row by different letters (a or b) significantly differ from each other (*p* < 0.05).

In the second series of experiments, 16 days after sowing, no significant difference in biomass was found between the variants (data not shown). However, after 32 days, the biomass of lettuce under coverings with LUM was 36 ± 3% higher than that of the reference covering (PP + PLA), and the photosynthesis increased by 30 ± 2% (Figures 6A,B). The respiration changed insignificantly and was 2.7 ± 0.1, 3.0 ± 0.2, and 2.4 ± 0.2 μmol CO_2_ m^−2^s^−1^ for the PP + PLA + LUM, PP + PLA, and PP + PLA + ABS coverings, respectively. At the same time, the leaf surface area remained practically unchanged (Figure 6C).

The cabbage biomass increased by 22 ± 2%, and photosynthesis increased by 45 ± 3% (Figures 6D,E). The respiration rate was 3.6 ± 0.3, 2.6 ± 0.2, and 3.0 ± 0.2 μmol CO_2_ m^−2^s^−1^ for PP + PLA+ LUM, PP + PLA, and PP + PLA + ABS, respectively. Thus, the use of the covering with the LUM enhances the respiration by 28 ± 4%. At the same time, the leaf area increases by 17% (Figure 6F).

The use of the covering with an organic absorbent (PP + PLA + ABS) having an absorption spectrum similar to that of PP + PLA + LUM made it possible to evaluate the effect of the luminescent component. Analysis of physiological parameters in plants grown under PP + PLA + ABS material showed no significant difference in the accumulation of total biomass, leaf surface area and photosynthesis rate in lettuce and cabbage plants compared to the reference PP + PLA covering (Figures 5, 6). Thus, the positive effect of the light-converting covering PP + PLA + LUM is associated with its luminescence in the red spectral region.

It should be noted that, in all series of experiments, the photosynthesis rate of lettuce and cabbage plants grown under the PP + PLA + LUM covering material is higher as compared to the other variants. Conversely, the value of stomatal conductance (Gs) in plants grown under the PP + PLA + LUM was smaller than in the reference experiment (PP + PLA; Table 2). Moreover, the usage of the covering in LUM led to a 3-fold reduction of the value of transpiration rate in both cabbage and lettuce. Eventually, the value of WUE increased markedly in plants under coating with LUM.

**Table 2:** The stomatal conductance, transpiration rate (Tr), photosynthesis rate, and water use efficiency (WUE) of 32-day-old cabbage and lettuce plants grown under the covering with the addition of photoluminophore (+LUM) and without the additive (–LUM).

Variant/parameters	Cabbage		Lettuce	
	PP + PLA + LUM	PP + PLA	PP + PLA + LUM	PP + PLA
Tr, mmol H_2_O, m^−2^s^−1^	1.72 ± 0.02^*^	5.51 ± 0.08	1.3 ± 0.01^*^	4.12 ± 0.09
G_s_, mol, m^−2^s^−1^	0.12 ± 0.01^*^	0.22 ± 0.02	0.10 ± 0.02^*^	0.165 ± 0.02
Pn, μmol CO_2_ m^−2^s^−1^	10.0 ± 0.8	8.2 ± 0.5	14.1 ± 1.2^*^	11.2 ± 0.9
WUE, μmol/mmol	6.0	1.6	10.9	2.7
Fv/Fm	0.80 ± 0.01	0.79 ± 0.02	0.81 ± 0.01	0.80 ± 0.01
F’v/F’m	0.53 ± 0.03	0.52 ± 0.04	0.56 ± 0.03	0.54 ± 0.02
Chl (*a* + *b*), mg/g (F.M.)	1.47 ± 0.08	1.36 ± 0.09	1.85 ± 0.11	1.72 ± 0.12
Carotenoids mg/g (F.M.)	0.24 ± 0.02	0.22 ± 0.02	0.37 ± 0.03	0.34 ± 0.03

Water use efficiency is equal to the ratio of the photosynthesis rate (Pn) to the transpiration rate. Fluorescence parameters [maximal *(Fv/Fm)* and effective *(F′v/F′m)* quantum yields] and the content of photosynthetic pigments were measured in 33-day-old plants. The average data for 10 leaves of plants in each variant are given.

* indicates a significant difference between PP + PLA and PP + PLA + LUM (*р* < 0.05).

The values are the means ± SD.

With insignificant changes in the respiration rate in lettuce plants, the balance of carbon dioxide absorption (difference between the rates of photosynthesis and respiration) was significantly higher in the experiments with the photoluminophore LUM: 6.0, 3.7, and 2.8 μmol CO_2_ m^−2^s^−1^ for the PP + PLA + LUM, PP + PLA, and PP + PLA + ABS series, respectively. Similar results for the photosynthesis minus respiration rates, but with a smaller difference between the variants, were obtained for cabbage: 4.5, 3.0, and 3.5 μmol CO_2_ m^−2^s^−1^ for the PP + PLA + LUM, PP + PLA, and PP + PLA + ABS series, respectively. This means that carbon uptake by leaves is maximized in the case of coverings containing LUM.

On the other hand, the presence of LUM in the covering led to a decrease in stomatal conductance and the leaf transpiration rate as well as to an increase in the WUE in both lettuce and cabbage plants (Table 2).

The efficiency of primary photosynthesis processes was estimated by measuring the fluorescence parameters: PSII maximum (*F*_v_/*F*_m_) and effective (*F*′_v_/*F*′_m_) quantum yields in lettuce and cabbage plants. The measurements of PSII effective (*F*’_v_/*F*’_m_) and the maximum (*F*_v_/*F*_m_) quantum yields showed that the presence in the covering of the photoluminophore LUM did not significantly affect these parameters, *F*_v_/*F*_m_ yield was in the range of 0.80 ± 0.01 on average and *F*′_v_/*F*′_m_ yield was 0.52 ± 0.04 on average for PP + PLA and PP + PLA + LUM variants, respectively (Table 2).

It was shown that in both cabbage and lettuce plants grown under PP + PLA + LUM and PP + PLA coverings there was a little difference in the content of Chl (*a* + *b*) and carotenoids (Table 2).

## Discussion

We found a noticeable increase in the photosynthesis rate and biomass accumulation in cabbage and lettuce seedlings when using coverings containing LUM, which is due to an increase in the fraction of orange-red light in the spectrum of solar radiation incident on the plants (Table 1). Moreover, this increase is not associated with a change in the absorption of the covering with the introduced photoluminophore, as seen from a similar experiment with the absorbent ABS. There are several mechanisms of the positive action of the PL introduced into the covering. One possible mechanism is the activation of the phytochrome system by luminescent red light emitted by a photoluminophore (Khramov et al., 2019). Luminescent RL in the spectrum of solar radiation incident on plants increases the RL/FRL ratio and, accordingly, the content of the active form of phytochrome. This can lead to an increase in the photosynthesis rate, stress resistance, and plant growth (Kasperbauer, 1988; Cao et al., 2018).

It has been shown that the introduction of an organic dye (Lumogen F-Red 300) into an agricultural polyethylene film to transform a part of light in the green–yellow spectral region to red light can be successfully used for growing tomato and rose plants (Novoplansky et al., 1990). The use of these materials as greenhouse coverings increased the yield of tomato fruits by 19.6% and the number of flowering branches on rose bushes by 26.7% compared to coverings without the fluorescent dye. A possible reason for the increased tomato yield is an enhancement of photosynthesis.

In some cases, blue fluorescent light can also have a stimulating effect on plants. For example, a positive effect of fluorescence in the blue region of the spectrum due to the introduction of fluorescent pigments into coatings on the productivity of strawberry plants has been demonstrated previously (Hemming et al., 2006). In that study, two types of fluorescent pigments were used: one type absorbs UV radiation and transforms it into blue light (410–480 nm) and the other type of pigments absorbs UV radiation and transforms it into orange-red radiation (610–630 nm or 600–690 nm). The authors concluded that only additional fluorescence in the blue spectral region (but not in the red region) leads to an increase in the productivity of strawberry plants.

Since the photosynthesis is sensitive to various components of the spectrum, including the RL region, the light of different wavelengths differently affects the light and dark stages of photosynthesis and, ultimately, biomass accumulation in plants (Bian et al., 2015; Martirosyan et al., 2019). Therefore, in our case, the redistribution of light energy from the region of 400–600 to 600–700 nm leads to a noticeable increase in photosynthesis but little affects respiration. It is likely that the enhancement of photosynthesis and respiration with increasing leaf area mainly leads to an increase in plant biomass, which is consistent with the increased accumulation of carbon in plant leaves, as estimated from the difference between CO_2_ uptake and release.

Primary light photochemical processes and the content of Chl affect the rate of photosynthesis. However, in our experiments, the luminescent RL does not influence the PSII activity (Table 2), i.e., the primary photochemical processes of photosynthesis. The Chl content changes also slightly. Therefore, the enhancement of photosynthesis is not due to the influence of luminescent RL on the primary photosynthetic processes or the Chl content. It can be assumed that RL affects photosynthesis through a change in the rate of CO_2_ uptake in chloroplasts. RL can change the stomatal conductance and the photosynthesis rate, usually increasing them (Boccalandro et al., 2009). However, in our experiments, stomatal conductance and the water evaporation rate are reduced in the series with the LUM-containing covering (Table 2), but this reduction does not lead to a decrease in the photosynthesis due to the reduced rate of CO_2_ uptake through the stomata. Apparently, stomatal conductance does not limit photosynthesis in this case. Most likely, the positive effect of LUM on the accumulation of biomass is explained by other reasons, first of all, by the energetic effect of luminescent RL, which increases photosynthesis and hence the growth of biomass. At the same time, with a significant increase in the RL fraction in the spectrum of light incident on plants, the contribution of the yellow-green light being a less effective for the photosynthesis as compared to the red and blue light decreases significantly (Figure 2; Table 1). Wherein, the share of BL in the radiation spectrum decreases simultaneously; however, according to the results of the experiments with an absorber, it can be concluded that the decrease in the share of BL with a simultaneous decrease in the share of yellow–green light does not significantly affect photosynthesis. Thus, the LUM luminescence in the red region of the spectrum plays a decisive role in the enhancement of photosynthesis.

Shortwave UV radiation inhibits photosynthesis (Kataria et al., 2013; Kosobryukhov et al., 2020). In our experiments, a decrease in the share of UV radiation in the spectrum of incident radiation could increase the photosynthesis rate in experiments with PLA. However, the fraction of shortwave UV radiation that passed through a greenhouse glass is little, and this factor plays an insignificant role.

In the plants of the LUM series, the energy-requiring water evaporation rate was found to be reduced (Table 2), and more energy could be expended for the photosynthesis and plant growth. At the same time, the accumulation of plant biomass increased due to an increase both in the photosynthesis rate and the leaf area. In lettuce plants with no noticeable increase in the leaf area, an increase in biomass is possible due to an increase in both leaf thickness and water content. However, the increase in carbon accumulation in leaves, observed under coverings with LUM, is more consistent with the fact that leaf biomass accumulates due to the increase in dry biomass and, consequently, the thickness of leaves.

The high WUE value found in leaves with LUM covering was surprising. Apparently, the increase in WUE is due to the presence of a large share of RL emitted by PL and falling into those places where just a small amount of solar RL can be absorbed by leaves (Khramov et al., 2019). Also, this may be one of the reasons for enhanced photosynthesis.

It should be noted that organic PLs such as LUM do not contain toxic components, and this is an important advantage as compared to the previously used metalloorganic PLs, which still contain moderately toxic rare-earth metals (Kusnetsov et al., 1989; Bratkova et al., 1998; Bratkova and Schelokov, 1999) and colloidal nanocrystals based on highly toxic cadmium (Simakin et al., 2020). Therefore, there are prerequisites for the practical application of covering textiles or films based on biodegradable polylactide materials containing organic PLs.

## Conclusion

The reproducible enhancement of plant growth due to the application of a light-converting polypropylene spunbond coated with the varnish based on PLA and LUM and having a high efficiency of conversion from the blue–green to orange–red light was shown. This leads to increased rate of photosynthesis and growth of cabbage and lettuce plants and the reduced water evaporation rate, which can be especially significant for yield sustainability in drought conditions. Therefore, there is a good reason to assume that the use of a great variety of covering materials (polymer films, glasses, varnishes, and textiles) containing organic PLs with characteristics similar to those of LUM can be promising in agrobiotechnology not only for green and vegetable crops but also for other field and greenhouse crops, algae cultivation in photobioreactors, and various woody and shrub crops, which requires further study. Thus, a novel light-converting agrotextile containing the photoluminescent PLA composition was developed, which opens up opportunities for the creation of biodegradable light-converting coverings for green technologies.

## Data Availability Statement

The original contributions presented in the study are included in the article/Supplementary Materials, further inquiries can be directed to the corresponding authors.

## Author Contributions

RK, VK, SP, and YL conceptualized the work. RK, VK, DB, and YL designed the experiments, analyzed the data, and wrote the article. RK, AKo, AKh, and VK performed the experiments with plants. ES and NS performed optical investigations. DB performed the synthesis. YL supervised the work. All authors contributed to the article and approved the submitted version.

## Funding

This project was supported by the Russian Foundation for Basic Research No 18-29-17073. NMR and UV–vis absorption and luminescence spectra were recorded using the equipment of Collaborative Access Center “Center for Polymer Research” of the Enikolopov Institute of Synthetic Polymeric Materials of the Russian Academy of Sciences under financial support from Ministry of Science and Higher Education of the Russian Federation (topic FFSM-2021-0005).

## Conflict of Interest

The reviewer IT declared a past co-authorship with one of the authors AK to the handling editor.

The remaining authors declare that the research was conducted in the absence of any commercial or financial relationships that could be construed as a potential conflict of interest.

## Publisher’s Note

All claims expressed in this article are solely those of the authors and do not necessarily represent those of their affiliated organizations, or those of the publisher, the editors and the reviewers. Any product that may be evaluated in this article, or claim that may be made by its manufacturer, is not guaranteed or endorsed by the publisher.

## Abbreviations

ABS: Absorbent
LUM: Photoluminophore used in this study
PL: Photoluminophore
PP: Polypropylene
PLA: Polylactide
PSII: Photosystem II
PAR: Photosynthetically active radiation
BL: Blue light
ORL: Orange–red light
YGL: Yellow–green light
RL: Red-light
FRL: Far-red light
