# Supplementary material for: Luminescence of Agrotextiles Based on Red-Light-Emitting Organic Luminophore and Polypropylene Spunbond Enhances the Growth and Photosynthesis of Vegetable Plants
Source: Front Plant Sci. 2022 Apr 21;13:827679. doi: 10.3389/fpls.2022.827679 (PMC9069102; doi:10.3389/fpls.2022.827679)
Supplement: Supplementary file 1 [file Data_Sheet_1.pdf]

ELECTRONIC SUPPORTING INFORMATION

to the Manuscript

Luminescence of agrotextiles based on red-light-emitting organic luminophore and polypropylene spunbond enhances the growth and photosynthesis of vegetable plants

Robert Khramov[1], Anatoly Kosobryukhov[2], Vladimir Kreslavski[2]*, Dmitry Balakirev[3], Alexandra Khudyakova[2], Evgeniya Svidchenko[3], Nikolay Surin[3], Sergey Ponomarenko[3], Yuriy Luponosov[3,4]*

[1]Institute of Theoretical and Experimental Biophysics, Russian Academy of Sciences, Pushchino, Moscow Region, Institutskaya 3, 142290, Russia

[2]Institute of Basic Biological Problems, Russian Academy of Sciences, Pushchino, Moscow Region, Institutskaya 2, 142290, Russia

[3]Enikolopov Institute of Synthetic Polymeric Materials of the Russian Academy of Sciences, Profsoyuznaya st. 70, Moscow, 117393, Russia

[4]Moscow State University, Chemistry Department, 1/3 Leninskie Gory, Moscow, 119991, Russia

**corresponding authors:* vkreslav@rambler.ru; luponosov@ispm.ru

1. Experimental part

1.1 Materials

2-Bromothiophene, [1,1′-bis(diphenylphosphino)ferrocene]dichloropalladium (II) (Pd(dppf)Cl_2), magnesium (0), *n*-butyllithium (2.5 M solution in hexane) (*n*-BuLi), 2-ethyl cyanoacetate, 3-(dicyanomethylidene)indan-1-one and polylactic acid (PLA) 4032D were purchased from Sigma-Aldrich Co. and used as-received. Chloroform, triethylamine, THF and DMF were purified and dried according to the known techniques. 7-[4-(Diphenylamino)phenyl]-2,1,3-benzothiadiazole-4-carboxaldehyde **(1)** and *N*-(4-bromophenyl)carbazole **(2)** were obtained as described elsewhere [1,2].

1.2 Synthetic procedures

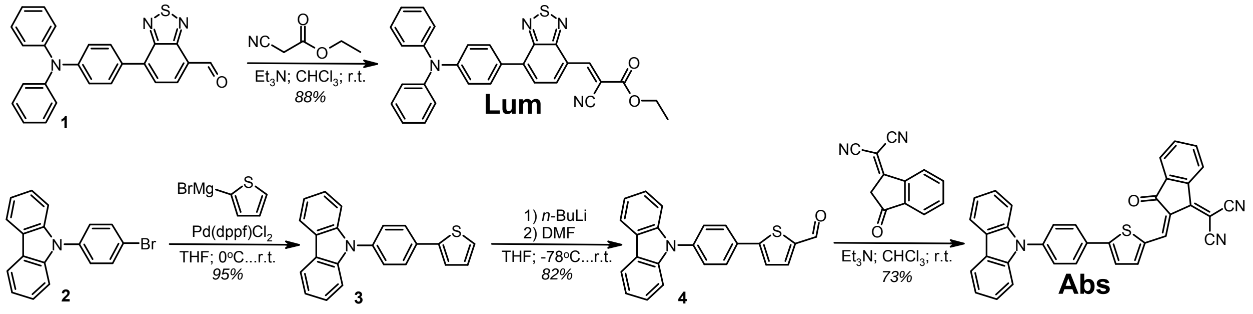

Figure S1. Synthesis scheme of organic luminophore Lum under discussion and nonluminescent model compound Abs.

Ethyl (2*E*)-2-Cyano-3-[7-[4-(diphenylamino)phenyl]-2,1,3-benzothiadiazol-4-yl]-2-propenoate (Lum) was obtained as described elsewhere [3] using compound **(1)** (2.00 g, 4.9 mmol) and 2-ethyl cyanoacetate (1.11 g, 9.8 mmol). The crude product was purified by column chromatography on silica gel (eluent: chloroform). Further purification included precipitation of the product from its THF solution with hexane to give pure product **Lum** (2.17 g, 88%) as bright red crystals. M.p. = 176 °C. ^{1}H NMR (250 MHz, $CDCl_3$): (ppm) 1.43 (t, 3H, J = 7.01 Hz); 4.42 (dd, 2H, J_1 = 14.34 Hz, J_2 = 7.02 Hz); 7.10 (t, 2H, J = 7.33 Hz); 7.14–7.23 (overlapping peaks, 6H); 7.31 (t, 4H, J = 7.32 Hz); 7.81 (d, 1H, J = 7.63 Hz); 7.92 (d, 2H, J = 8.85 Hz); 8.84 (d, 1H, J = 7.63 Hz); 9.25 (s, 1H). Calcd (%) for $C_{30}H_{22}N_4O_2S$: C, 71.69; H, 4.41; N, 11.15; S, 6.38. Found C, 71.71; H, 4.42; N, 11.16; S, 6.38. MALDI-MS: found m/z 502.0; calculated for $[M]^+$ 502.1.

9-[4-(2-Thienyl)phenyl]-9*H*-carbazole (3). A solution of 2-bromothiophene (5.23 g, 32.1 mmol) in 50 mL of anhydrous THF was added dropwise to the suspension of magnesium turnings (0.80 g, 33.0 mmol) in 5 mL of anhydrous THF. The Grignard reagent was refluxed for 1 h and

then cooled to room temperature. Afterwards *in situ* prepared solution of Grignard reagent was slowly added to the solution of compound **(2)** (7.75 g, 24.1 mmol) and Pd(dppf)Cl_2 (117 mg, 0.2 mmol) in 50 mL of anhydrous THF at 0°C. The temperature was then allowed to rise to room temperature and then the reaction was stirred for another 12 h. After reaction was completed, 300 mL of freshly distilled diethyl ether and 100 mL of distilled water were added to the reaction mixture. The organic phase was separated and washed with water. The solvent was evaporated under vacuum and the crude product was purified by a column chromatography on silica gel (eluent: toluene) with following recrystallization from toluene:hexane mixture to give pure product **3** (7.44 g, 95 %) as a colorless solid. ^{1}H NMR (250 MHz, $CDCl_3$): (ppm) 7.12 (dd, 1H, J_1 = 5.19 Hz, J_2 = 3.66 Hz); 7.25–7.48 (overlapping peaks, 8H); 7.56 (d, 2H, J = 8.54 Hz); 7.82 (d, 2H, J = 8.85 Hz); 8.14 (d, 2H, J = 7.63 Hz). Calcd (%) for $C_{22}H_{15}NS$: C, 81.20; H, 4.65; N, 4.30; S, 9.85. Found C, 81.22; H, 4.65; N, 4.29; S, 9.84.

5-[4-(9*H*-Carbazol-9-yl)phenyl]-2-thiophenecarboxaldehyde (4). *n*-BuLi (2.5 M solution in hexane) (2.5 mL, 6.1 mmol) was added dropwise to a solution of compound **(3)** (2.00 g, 6.1 mmol) in 55 mL of anhydrous THF at –78°C. Afterwards, the reaction mixture was stirred for 60 min at –78°C. Then dry DMF (0.45 g, 6.2 mmol) was added by one portion to the solution at –78 °C. The reaction mixture was stirred for 30 min at –78°C, then the cooling bath was removed, and the stirring was continued for another 30 min with raising the temperature to room temperature. The reaction mixture was poured into distilled water (150 mL) and neutralized with 1 M HCl (6 mL), and then extracted with freshly distilled diethyl ether (300 mL) twice. The combined organic phases were dried over sodium sulfate and filtered. The solvent was evaporated and the crude product was purified by column chromatography on silica gel (eluent: dichloromethane) to give pure product **4** (1.78 g, 82 %) as a pale yellow solid. ^{1}H NMR (250 MHz, $CDCl_3$): (ppm) 7.31 (t, 2H, J = 7.63 Hz); 7.38–7.52 (overlapping peaks, 5H); 7.64 (d, 2H, J = 8.55 Hz); 7.79 (d, 1H, J = 3.96 Hz); 7.88 (d, 2H, J = 8.55 Hz); 8.14 (d, 2H, J = 7.63 Hz); 9.93 (s, 1H). Calcd (%) for $C_{23}H_{15}NOS$: C, 78.16; H, 4.28; N, 3.96; S, 9.07. Found C, 78.15; H, 4.28; N, 3.96; S, 9.08.

[(2*E*)-2-({5-[4-(9*H*-carbazol-9-yl)phenyl]-2-thienyl}methylene)-3-oxo-2,3-dihydro-1*H*-inden-1-ylidene]malononitrile (Abs). Compound (**4**) (0.53 g, 1.5 mmol), 3-(dicyanomethylidene)indan-1-one (0.32 g, 1.7 mmol) and dry pyridine (20 mL) were placed in a reaction vessel and stirred under an argon atmosphere for 1 hours at r.t. After the reaction completion, pyridine was evaporated under vacuum and the residue was dried at 1 Torr. The crude product was purified by column chromatography on silica gel (eluent: dichloromethane). Further

purification included precipitation of the product from its THF solution with hexane to give pure product **Abs** as a black solid (0.58 g, 73%). ^{1}H NMR (250 MHz, $CDCl_3$): (ppm) ^{1}H NMR (250 MHz, $CDCl_3$): (ppm) 7.31 (t, 2H, J = 7.63 Hz); 7.38–7.52 (overlapping peaks, 4H); 7.58 (d, 1H, J = 4.27 Hz); 7.68 (d, 2H, J = 8.85 Hz); 7.78 (t, 2H, J = 6.11 Hz); 7.89–7.99 (overlapping peaks, 2H); 8.02 (d, 2H, J = 8.55 Hz); 8.14 (d, 2H, J = 7.32 Hz); 8.70 (d, 1H, J = 7.32 Hz); 8.90 (s, 1H). Calcd (%) for $C_{35}H_{19}N_3OS$: C, 79.38; H, 3.62; N, 7.93; S, 6.05. Found C, 79.40; H, 3.63; N, 7.92; S, 6.05. MALDI-MS: found m/z 529.1; calculated for $[M]^+$ 529.1.

1.3. Characterization

NMR spectra. ^{1}H NMR spectra were recorded in a “Bruker WP-250 SY” spectrometer, working at a frequency of 250.13 MHz and using $CDCl_3$ signal (7.25 ppm) as the internal standard. The compounds to be analyzed were taken in the form of 1% solutions in $CDCl_3$. The spectra were then processed on the computer using the “ACD Labs” software.

Elemental analysis. Elemental analysis of C, N and H elements was carried out using CHN automatic analyzer “CE 1106” (Italy). The settling titration using $BaCl_2$ was applied to analyze Sulphur.

Mass-spectra. Mass-spectra (MALDI-TOF) were registered on the “Autoflex II Bruker” (resolution FWHM 18000), equipped with a nitrogen laser (work wavelength 337 nm) and time-of-flight mass-detector working in reflections mode. The accelerating voltage was 20 kV. Samples were applied to a polished stainless-steel substrate. Spectrum was recorded in the positive ion mode. The resulting spectrum was the sum of 300 spectra obtained at different points of sample. 2,5-Dihydroxybenzoic acid (DHB) (Acros, 99%) and α-cyano-4-hydroxycinnamic acid (HCCA) (Acros, 99%) were used as matrices.

2. NMR spectra

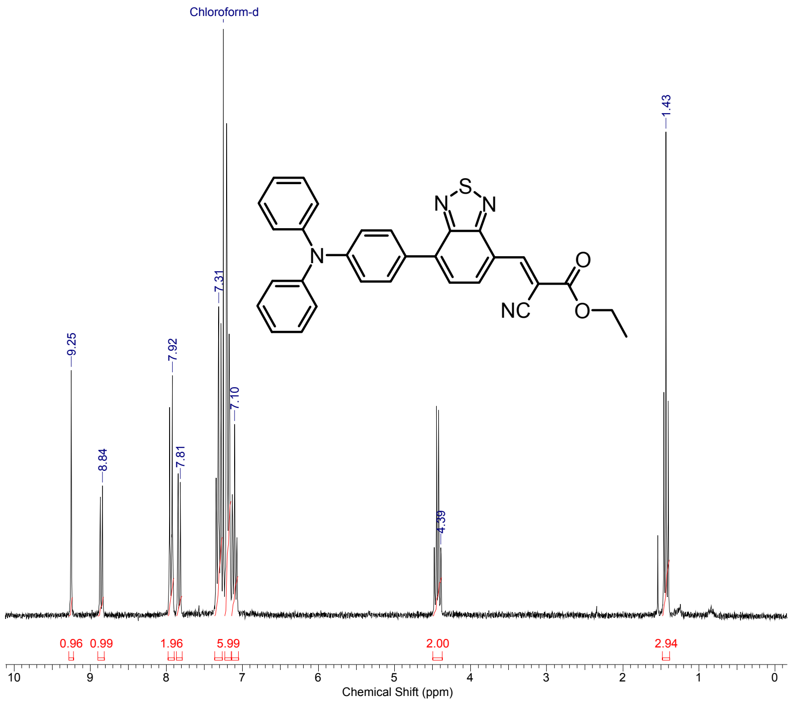

No.	Annotation	(ppm)
1	Chloroform-d	7.25

No.	(ppm)	Value	Absolute Value
1	[1.38 .. 1.48]	2.935	3.25129e+7
2	[4.37 .. 4.50]	2.004	2.22009e+7
3	[7.05 .. 7.14]	2.052	2.27269e+7
4	[7.15 .. 7.23]	5.986	6.62980e+7
5	[7.26 .. 7.36]	4.083	4.52285e+7
6	[7.79 .. 7.87]	1.030	1.14084e+7
7	[7.90 .. 7.98]	1.960	2.17085e+7
8	[8.82 .. 8.90]	0.990	1.09691e+7
9	[9.22 .. 9.28]	0.960	1.06376e+7

Figure S2. ^{1}H NMR spectrum of **Lum**

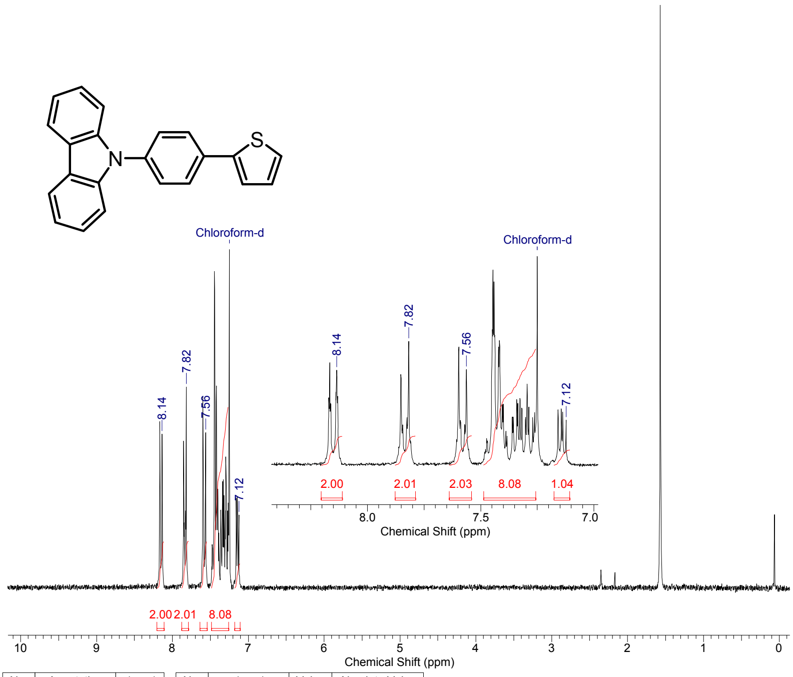

No.	Annotation	(ppm)
1	Chloroform-d	7.25

No.	(ppm)	Value	Absolute Value
1	[7.11 .. 7.18]	1.042	7.87257e+6
2	[7.26 .. 7.49]	8.076	6.10299e+7
3	[7.54 .. 7.64]	2.033	1.53630e+7
4	[7.79 .. 7.88]	2.008	1.51725e+7
5	[8.11 .. 8.20]	2.000	1.51148e+7

Figure S3. ^{1}H NMR spectrum of compound **3**

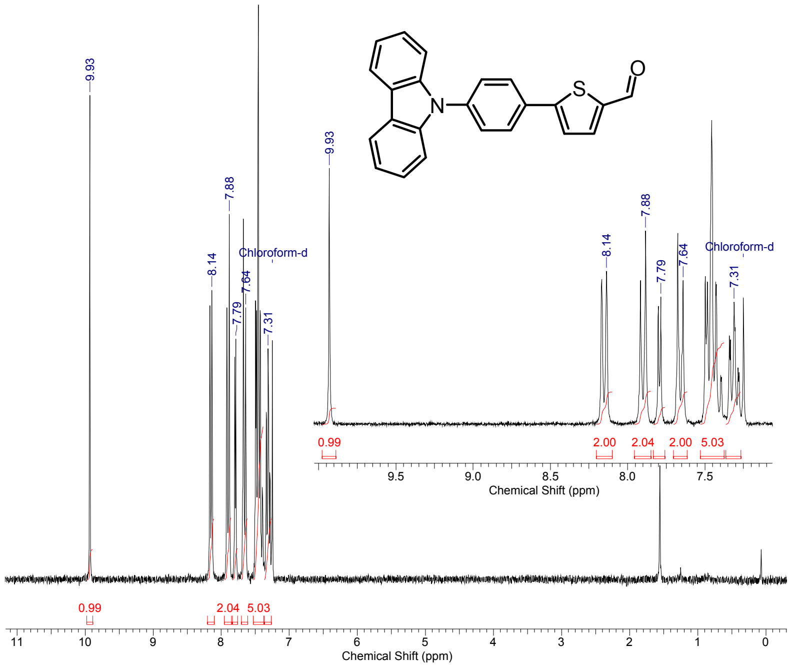

No.	Annotation	(ppm)
1	Chloroform-d	7.25

No.	(ppm)	Value	Absolute Value
1	[7.27 .. 7.36]	2.001	4.88012e+7
2	[7.38 .. 7.53]	5.033	1.22761e+8
3	[7.61 .. 7.70]	2.005	4.88986e+7
4	[7.76 .. 7.83]	1.036	2.52653e+7
5	[7.85 .. 7.96]	2.038	4.97107e+7
6	[8.10 .. 8.20]	2.000	4.87802e+7
7	[9.89 .. 9.98]	0.995	2.42614e+7

Figure S4. ^{1}H NMR spectrum of compound **4**

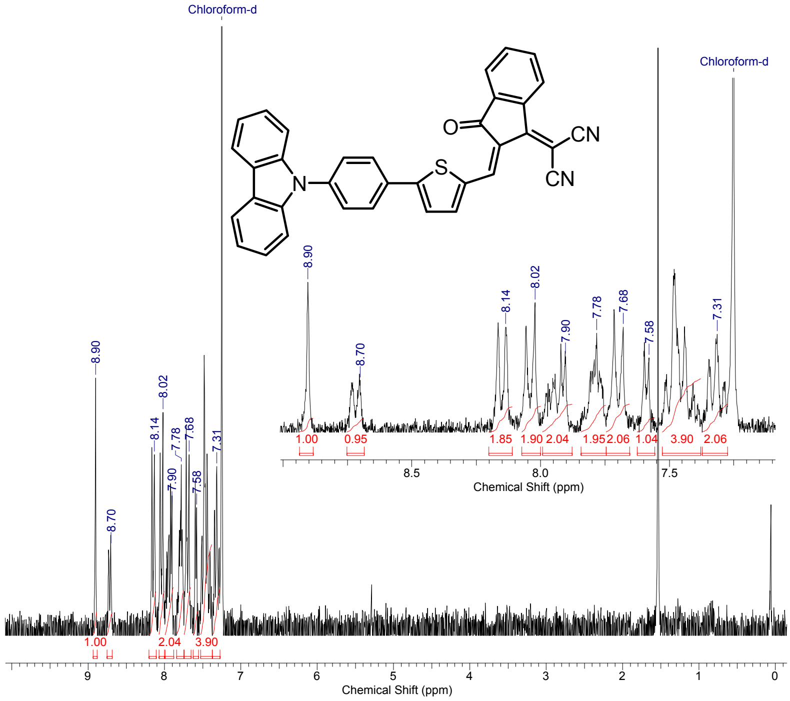

No.	Annotation	(ppm)
1	Chloroform-d	7.25

No.	(ppm)	Value	Absolute Value
1	[7.27 .. 7.37]	2.062	1.67921e+7
2	[7.38 .. 7.53]	3.903	3.17801e+7
3	[7.56 .. 7.62]	1.040	8.47104e+6
4	[7.65 .. 7.75]	2.058	1.67587e+7
5	[7.75 .. 7.84]	1.954	1.59142e+7
6	[7.88 .. 7.99]	2.042	1.66240e+7
7	[8.00 .. 8.07]	1.895	1.54344e+7
8	[8.11 .. 8.20]	1.847	1.50364e+7
9	[8.69 .. 8.75]	0.950	7.73248e+6
10	[8.88 .. 8.94]	1.000	8.14282e+6

Figure S5. ^{1}H NMR spectrum of **Abs**

References

[1] Z. Sun, Q. Zang, Q. Luo, C. Lv, F. Cao, Q. Song, R. Zhao, Y. Zhang, W. Wong. Chem. Commun., 2019, 55, 4735-4738

[2] T. Ramsingh Girase, S. Bhilare, S. Sankar Murthy Bandaru, N. Chrysochos, C. Schulzke, Y. S. Sanghvi, A. R. Kapdi, Asian J. Org. Chem. 2020, 9, 274.

[3] D.-H. Roh, K. M. Kim, J. Seung Nam, U.-Y. Kim, B.-M. Kim, J. S. Kim, T.-H. Kwon. J. Phys. Chem. C 2016, 120, 43, 24655–24666
